# Biomarkers of early stage osteoarthritis, rheumatoid arthritis and musculoskeletal health

**DOI:** 10.1038/srep09259

**Published:** 2015-03-19

**Authors:** Usman Ahmed, Attia Anwar, Richard S. Savage, Matthew L. Costa, Nicola Mackay, Andrew Filer, Karim Raza, Richard A. Watts, Paul G. Winyard, Joanna Tarr, Richard C. Haigh, Paul J. Thornalley, Naila Rabbani

**Affiliations:** 1 Warwick Medical School, Clinical Sciences Research Laboratories, University of Warwick, University Hospital, Coventry CV2 2DX, U.K.; 2 Warwick Systems Biology Centre, Coventry House, University of Warwick, Coventry CV4 7AL, U.K.; 3 Sandwell and West Birmingham Hospital NHS Trust, Dudley Road, Birmingham B18 7QH, West Midlands, U.K.; 4 Centre for Translational Inflammation Research, University of Birmingham, Birmingham B15 2TT, U.K.; 5 Ipswich Hospital NHS Trust, Ipswich IP4 5PD, Suffolk, U.K.; 6 Medical School, University of East Anglia, Norwich, NR4 7TJ U.K.; 7 University of Exeter Medical School, St Luke's Campus, Exeter EX1 2LU, U.K.; 8 Department of Rheumatology, Royal Devon and Exeter NHS Foundation Trust, Exeter, U.K.; 9 Warwick Clinical Trials Unit, University of Warwick, Coventry CV4 7AL, U.K.

## Abstract

There is currently no biochemical test for detection of early-stage osteoarthritis (eOA). Tests for early-stage rheumatoid arthritis (eRA) such as rheumatoid factor (RF) and anti–cyclic citrullinated peptide (CCP) antibodies require refinement to improve clinical utility. We developed robust mass spectrometric methods to quantify citrullinated protein (CP) and free hydroxyproline in body fluids. We detected CP in the plasma of healthy subjects and surprisingly found that CP was increased in both patients with eOA and eRA whereas anti–CCP antibodies were predominantly present in eRA. A 4-class diagnostic algorithm combining plasma/serum CP, anti-CCP antibody and hydroxyproline applied to a cohort gave specific and sensitive detection and discrimination of eOA, eRA, other non-RA inflammatory joint diseases and good skeletal health. This provides a first-in-class plasma/serum-based biochemical assay for diagnosis and type discrimination of early-stage arthritis to facilitate improved treatment and patient outcomes, exploiting citrullinated protein and related differential autoimmunity.

Musculoskeletal disease including osteoarthritis (OA) and rheumatoid arthritis (RA) is the most common cause of chronic disability worldwide and is increasingly important in current ageing populations. It is a major contributor to global disability adjusted life years^1^. Severe life impairment may be prevented if decline in musculoskeletal health and development of OA and RA are identified and treated in the early stages. An inexpensive, minimally invasive biochemical test which preferably detects and distinguishes common types of arthritis at the early stage is required. Magnetic resonance imaging (MRI) techniques have been developed for early-stage evaluation of cartilage damage in OA. They have approximately 70% sensitivity and 90% specificity compared to reference diagnosis by arthroscopy, thus lending more utility to excluding OA diagnosis when otherwise suspected than detecting new OA^2^. MRI techniques require expensive instrumentation time and facilities as well as being contraindicated in certain populations who have implanted devices such as pacemakers or aneurysm coils. Early biochemical tests for detection of established RA were based on measurement of RF which in current form have reported sensitivity and specificity of 63% and 94% respectively for established or advanced disease^3^. RF is often negative with eRA. The anti-CCP antibody test is used for early-stage detection of RA and has sensitivity of 61%^4^. There is currently no simple biochemical test to detect eOA and to discriminate different types of early-stage arthritis.

The clinical presence of anti-CCP antibodies, antibodies which bind to synthetic cyclic citrullinated peptide, are considered to reflect immunogenicity of endogenous citrullinated proteins (CPs) but the diagnostic utility of CPs has hitherto been little explored. The formation of citrulline residues in proteins occurs by a post-translational modification of arginine residues catalysed by members of the peptidylarginine deiminase (PAD) family of enzymes^5^ (Figure 1a). This process is considered to be a marker of inflammation. CPs are immunogenic *in vivo* and are involved in autophagic presentation of antigens^5^. A further biochemical marker historically linked to skeletal health and disease is 4-hydroxyproline (Hyp). Plasma Hyp is considered to be a marker of bone turnover and resorption^6^. Recent clinical studies suggest 62% of the variation in plasma free Hyp relates to bone metabolism^7^. We hypothesised that changes in plasma CP and Hyp, combined with anti-CCP antibody test, would provide improved diagnostic power over current standard techniques for diagnosis of early-stage arthritis. Methods for robust quantitation of CP and Hyp are currently lacking. To test our hypothesis we developed a robust stable isotopic dilution analysis mass spectrometric method for the detection of CP and Hyp.

We describe herein robust quantitation of total CP and free Hyp and show that CP is surprisingly present at high levels in both eOA and eRA, and when estimates of CP and plasma Hyp are combined with the anti-CCP antibody test in a diagnostic algorithm using machine learning techniques, early-stage arthritic diseases from control group can be readily detected and distinguished.

## Results

**Figure 1:**
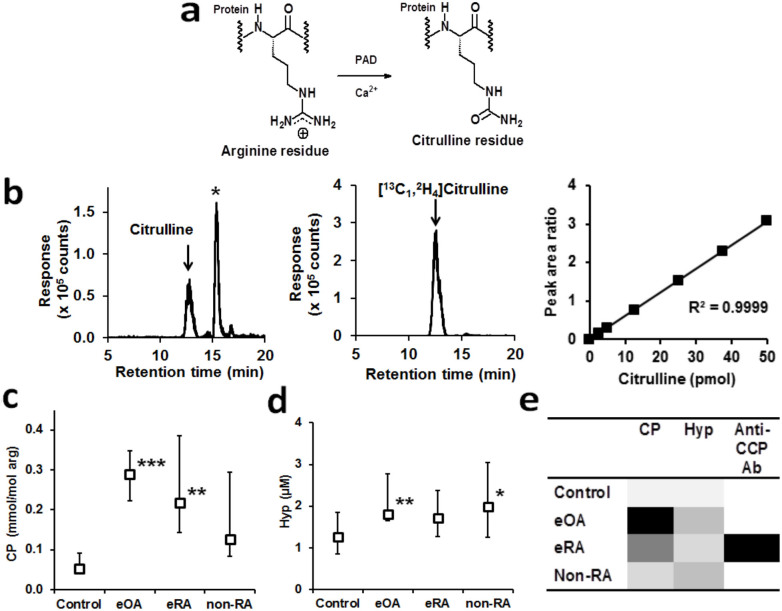
(a) Formation of citrullinated protein. (b) Analysis of citrullinated protein. Detection and quantitation of citrulline by stable isotopic dilution analysis LC-MS/MS. *Left-hand panel* – MRM chromatogram of citrulline in plasma protein digest from patient with eOA, *middle panel* - MRM chromatogram of [5-^13^C-4,4,5,5-^2^H_4_]citrulline internal standard (5 pmol), *right-hand panel* – calibration curve of citrulline/[5-^13^C-4,4,5,5-^2^H_4_]citrulline MRM chromatographic peak area ratio against citrulline standard (pmol). (c) Citrullinated protein (CP) and (d) hydroxyproline (Hyp) in early-stage arthritis and healthy human subjects. Samples were plasma for Control (n = 16) and eOA (n = 16) and serum for eRA (n = 10) and non-RA (n = 10). Significance: *, ** and ***, *P* < 0.05, *P* < 0.01 and *P* < 0.001 with respect to control (healthy people). Data are median (lower – upper quartile). (e) Discrimination of study groups by discrete patterns of CP, Hyp and Anti-CCP antibody response. Each darkened shade presents approximately a 2-fold increase.

We developed a stable isotopic dilution analysis liquid chromatography-tandem mass spectrometry (LC-MS/MS) protocol for the quantitation of total CP in plasma. Plasma, serum or synovial fluid protein is separated from free citrulline by microspin diafiltration, digested to component amino acids by exhaustive enzymatic hydrolysis and citrulline released from CPs quantified. CP contents of plasma or serum protein are normalised to arginine content and given as mmol/mol arg. The method had high specificity, good linearity of response from 62–50,000 fmol (R^2^ = 0.9999) and high sensitivity - the limit of detection was 62 fmol, equivalent to 0.006 mmol/mol arg in plasma protein under assay conditions (Figure 1b and Supplementary Table S1). CP was detected and quantified in plasma protein of healthy people. Plasma CP was 0.053 (0.043–0.091) mmol/mol arg (n = 16) and correlated positively with plasma Hyp (r = 0.63, P < 0.05). CP was increased 4-fold in serum protein of patients with eRA and surprisingly was even higher, increased 5-fold, in patients with eOA, with respect to healthy controls. CP was not increased in serum of patients with inflammatory arthritis other than RA (non-RA) (Figure 1c). There was no association of increased CP with smoking (ever versus never smoked), inflammatory markers (plasma/serum C-reactive protein concentration and erythrocyte sedimentation rate) or alcohol consumption. Herein CP content of serum samples for eRA and non-RA are compared with plasma of other study groups. Serum is comparable to plasma as a sample matrix as the major protein lost during clot formation, fibrinogen, has relatively low citrulline content and concentration in plasma^8^, equivalent to <0.001 mmol/mol arg in CP analysis herein. Replicate analyses gave a mean estimate range of 14% (n = 12) and Bland-Altman plot indicated no proportional bias.

Free Hyp was analysed in plasma, serum and synovial fluid by stable isotopic dilution analysis LC-MS/MS. The method had high specificity, good linearity of response from 0.1–50 pmol and high sensitivity (R^2^ = 0.9997). The limit of detection was 102 fmol (Supplementary Table S1). The median concentration of Hyp in plasma of healthy people was 1.26 (0.86–1.86) μM. Plasma Hyp was increased 44% in eOA and 58% in non-RA but not increased in eRA with respect to healthy controls (Figure 1d). Most patients with eRA were positive for anti-CCP antibodies whereas healthy controls, eOA and non-RA were all negative. For eRA, plasma CP in patients positive for anti–CCP antibodies was significantly higher than plasma CP of healthy controls - 0.259 (0.186–0.607) mmol/mol arg, *P* < 0.01 but not in patients negative for anti–CCP antibodies. By this variation of analytes - plasma/serum CP, Hyp and anti-CCP antibody positivity, a distinctive analyte pattern emerged for each study group (Figure 1e). This is exploited in a diagnostic algorithm developed using a machine learning analysis approach. Replicate analyses gave a mean estimate range of 15% (n = 12) and Bland-Altman plot indicated no proportional bias.

**Figure 2:**
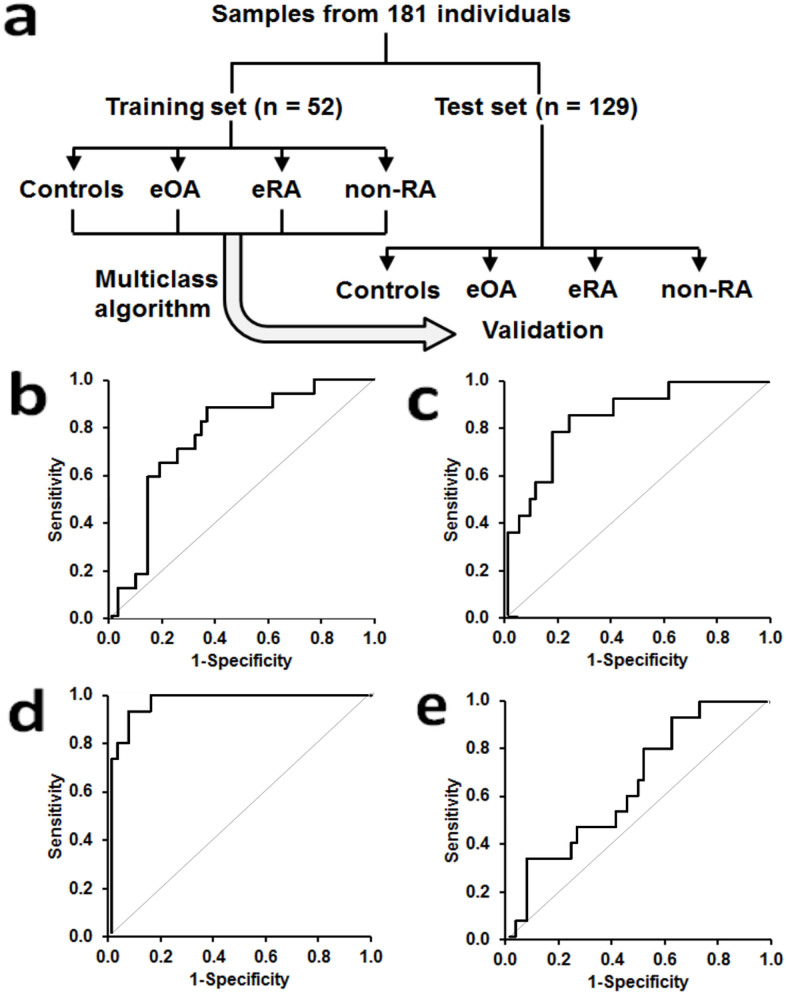
Training and validation of a multiclass algorithm for detection and discrimination of early stage osteoarthritis, rheumatoid arthritis, other inflammatory joint disease from healthy subjects. (a) Training set and test set study groups. Receiver operating characteristic curves in the test set for detection of: (b) healthy controls (n = 37), (c) eOA (n = 30), (d) eRA (n = 35) and (e) non-RA (n = 32).

We performed a machine learning analysis on subject groups with and without early-stage arthritis to assess the predictive power of the measured biomarkers. In all cases, the predictive algorithms were trained on the training data set, before being used to predict the disease class for each sample in the test data set (Figure 2a). The objective was to distinguish simultaneously between four groups: healthy control, eOA, eRA and non-RA – particularly useful in the clinical context. We took two approaches: firstly, we used a set of 4 one-versus-all classification tasks, and secondly we used a single overall analysis using multinomial logistic regression. In the one-versus-all analyses, we ran Random Forests^9^ and GLMNET classification algorithms^10^, as well as an ensemble^11^ that combined both of these in equal proportions. We also considered Gaussian process, Generalised Boosting model, and K-nearest-neighbour algorithms but found these to be no better than GLMNET, so we do not report those results here. In all analyses, we use the panel of three biomarkers given above, plasma/serum CP, Hyp and anti-CCP antibody positivity, and subject age and gender. The outcome of each analysis was to assign, for each test set sample, a set of four probabilities corresponding to each of the four disease/control groups. The predicted group is then the one for which the probability is highest. The test data were held separate from the algorithm training and no algorithm settings were adjusted after generating the test set results – providing for a rigorous estimate of predictive performance on previously unseen cases. We performed Leave-One-Out (LOO) cross-validation analyses, comparing in turn each of the classes with the other three, thereby providing estimation of the predictive performance of each machine learning algorithm, when trained on the training set data. The clinical characteristics of training and test set study groups are given in Table 1. GLMNET algorithm gave the best outcome through training and test sets. Applying the algorithm for 4 classes, for which random assignment sensitivity and specificity = 0.25, the GLMNET algorithm gave sensitivities of 0.41–0.73 for groups excluding non-RA and specificities of 0.75–0.91 for all groups and highest combined F-measure (*P* = 3.5 × 10^−7^) in the test set validation – Table 2. The outcome is presented also as a confusion matrix – Table 3. A limitation of the study was relatively small numbers of cases on the eRA and non-RA study groups in the training set. To assess the impact of this, further cross validation was performed whereby the training and test set subjects were combined and then 67% of the subjects from each group were selected randomly as a surrogate training set and the remaining 33% of subjects as a surrogate test set. The re-assessed GLMNET analysis gave improved diagnostic performance - sensitivities of 0.67–0.76 for groups excluding non-RA and specificities of 0.77–1.00 for all groups and highest combined F-measure (*P* = 2.9 × 10^−11^), confirming and improving the security of the 4-class diagnosis outcomes – Table S2. Receiver operating characteristic (ROC) curves gave area under curves (mean (95% confidence intervals): healthy control 0.77 (0.64–0.90), eOA 0.86 (0.75–0.97), eRA 0.98 (0.95–1.00), and non-RA 0.65 (0.50–0.81) – Figure 2, b.–d.

**Figure 3:**
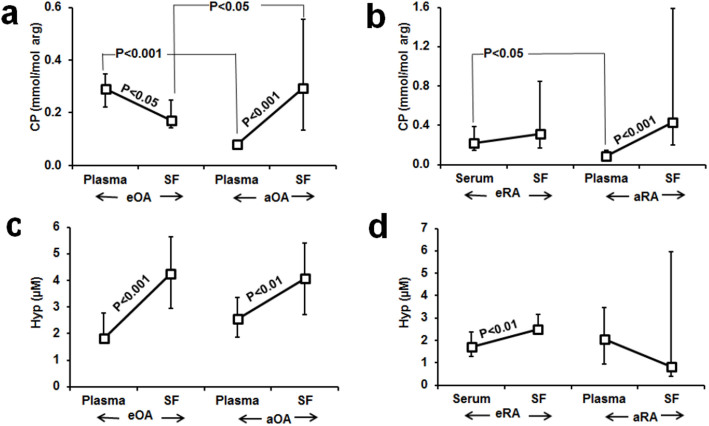
Citrullinated protein and hydroxyproline in plasma and synovial fluid compartments in early-stage and advanced arthritis. CP: (a) osteoarthritis and (b) rheumatoid arthritis. Hyp: (c) osteoarthritis and (d) rheumatoid arthritis. Data are median (lower – upper quartile); for eOA, n = 16; aOA, n = 17, eRA, n = 10 and aRA, n = 22. Lines joint median estimates in plasma/serum to synovial fluid from the same subject groups.

We also compared CP and Hyp in plasma or serum and synovial fluid of early and advanced stages of OA and RA. In eOA, there was a negative gradient of CP from plasma into synovial fluid whereas in aOA this was reversed and there was a strong positive gradient from plasma to synovial fluid. Plasma CP was decreased 73% in aOA, compared to plasma CP in eOA whereas synovial fluid CP was increased in 72% in aOA compared to synovial fluid CP in eOA (Figure 3a). In eRA, there was no significant difference in CP content of serum and synovial fluid whereas in aRA there was a strong positive gradient from plasma to synovial fluid. Plasma CP was decreased 59% in aRA, compared to serum CP in eRA whereas plasma and synovial fluid CP in aRA were similar levels (Figure 3b). Serum CP correlated positively with synovial fluid CP in patients with eRA (r = 0.81, *P* < 0.05) but not in patients with aRA. There were strong positive gradients of Hyp from plasma to synovial fluid in both eOA and aOA but no significant difference of plasma or synovial fluid Hyp in aOA compared to eOA (Figure 3c). In eRA there was a strong positive gradient of Hyp from serum to synovial fluid but not in aRA (Figure 3d). Plasma Hyp correlated positively with Hyp in synovial fluid in eOA, r = 0.70, P < 0.01 and aOA, r = 0.56, *P* < 0.05; and serum Hyp correlated positively with synovial fluid Hyp in eRA (r = 0.88, P < 0.001).

## Discussion

Detecting and distinguishing different types of early-stage arthritis is unachievable routinely – if attempted, it requires arthroscopy or MRI for detection of eOA in combination with immunochemical tests to distinguish eRA. There is no clinically established method to detect eOA; radiography remains the method of choice for staging OA but shows little or no changes in eOA. Hence when OA is confirmed significant pain, discomfort and damage to the joint is already present. Diagnosis of eRA is made by immunochemical tests, often before damage to the joint occurs. The diagnostic outcome described herein, combination of plasma/serum CP, Hyp and anti-CCP antibody in a diagnostic algorithm, is therefore a first-in-class technique providing for biochemical diagnosis and discrimination of eOA, eRA and non-RA and healthy controls. It requires minimally invasive, non-expert sampling and analysis, and has relatively high throughput and low cost. Biochemical tests for the detection of eOA have been proposed previously^12^ but our test provides both early detection and discrimination of type of arthritis by exploiting the CP epitope shown herein for the first time to be common in relatively high abundance in both early-stage OA and RA with autoimmunity restricted to early-stage RA. The strengths of using the machine learning approach in producing the diagnostic algorithm are that it provides a data-driven discovery of complex combinations of independent variables or features and their relative influence on the dependent variable of interest (diagnosis of early-stage type of arthritis) by logical, step-wise analysis without preconception and bias. The limitations are access to restricted application specific data – variables recorded and dataset size, and as a post hoc analysis all data collection must be completed before implementation. It also requires expertise for awareness of variables available for inclusion in the analysis – which applies to other types of data analysis.

The surprising and remarkable biochemical finding of this study is increased levels of plasma CP in eOA. Increased CP was found in serum of eRA but this was suspected with regard to the high prevalence of anti-CCP antibody positivity in eRA. Also remarkable was the higher CP concentration in plasma than in synovial fluid of patients with eOA and reversal of this in patients with aOA. Recent research has suggested movement of plasma proteins into the synovium occurs in eOA and contributes to an inflammatory response^13^. It is now accepted that there is an inflammatory component of OA with a decrease in inflammatory mediators in advanced disease^14^. Inflammatory mechanisms linked to CP formation may be a component of this and compartmentalised between the vasculature and synovium. PAD2 and PAD4 were detected in synovial tissue of patients with OA^15^ and dysfunction of autophagy has been linked to cartilage destruction in OA^16^. Multi-compartmental expression and activity of PADs, plasma and synovial fluid CPs and links to dysfunctional autophagy and cartilage loss now require investigation.

Autoimmunity to CP is important in the pathogenesis of RA and underlies the diagnostic utility of anti-CCP antibody measurement for eRA^5^. We found high levels of serum CP in patients with eRA and association of this with anti-CCP antibodies, consistent with formation and immunogenicity of CPs. In eRA, similar concentrations of CP in serum and synovial fluid are consistent with equilibration of CP between plasma and synovial fluid *in vivo* and/or similar factors influencing CP formation in both compartments. PAD2 and PAD4 enzymes have been detected in synovial tissue and also lymphocytes and monocytes in patients with RA^15^ and are likely drivers for CP formation in RA. The lack of association of CP with smoking in eRA may be due to the relatively old age of onset of RA studied – mean age of 65 yrs in the training set and 60 yrs in the test set. Recent risk prediction models of large cohort studies indicate that for onset of RA at age 60–65 the risk of developing RA is similar in never and ever smokers in both males and females^17^.

The presence of CPs in plasma of healthy people implicates PADs in pre-symptomatic low grade inflammation which may be activated and increased in susceptible individuals and environments. Autoimmunity to several CPs has been detected prior to development of RA^18^ and is linked to bone resorption^19^. The correlation of CP with Hyp in plasma of healthy subjects found herein may betray a link of protein citrullination to bone resorption of pre-symptomatic arthropathy.

The detection and quantitation of CP in plasma/serum, synovial fluid and elsewhere poses a significant analytical challenge. The citrulline residue epitope in proteins occurs in trace amounts (0.1–0.01% arginine residues) in the presence of 10–50 fold higher concentration of free, dialyzable citrulline - a metabolite of the urea cycle and co-product of nitric oxide synthases^20^. The reference method for quantitation of trace amino acid residues such as citrulline residues in CPs is hydrolysis of proteins to component amino acids and quantitation by stable isotopic dilution analysis LC-MS/MS. Enzymatic digestion is used to avoid compromise of analyte content by harsh conditions for hydrolysis during pre-analytic processing^21^. The analysis of CP herein provides an estimate of total CP – the sum of citrulline residues in all proteins of plasma or synovial fluid. Individual CPs have been identified by mass spectrometry proteomics but approaches to date have lacked robust quantitation and mass spectrometric detection of the citrulline residue^22^. Stable isotope-dilution mass spectrometry methods such as this are considered reference methods of extremely high accuracy as they have high specificity to analyte for detection response and all sample matrix-related effects affect target analyte and stable isotope-labelled internal standard in a similar manner^23^. The assay has good day-to-day reproducibility and sample stability during batch analysis.

Robust measurement of free Hyp in plasma was also achieved herein. This gave lower estimates in plasma and synovial fluid than those using other techniques^24^,^25^,^26^, which is likely due to the high specificity of stable isotopic dilution analysis LC-MS/MS.

Limitations of the study are the requirement for further clinical validation of the diagnostic algorithm and the cross-sectional design of the study. In further studies validation with larger subject groups and evaluation of the link of the diagnostic output to progression to advanced-stage arthritis are important issues to address. Selection for eOA subjects from subjects with new onset knee pain and Outerbridge classification low grade changes and joint changes on arthroscopic investigation consistent with eOA may have included some subjects with other features – such as synovial inflammation (found in some eOA^27^) or bone marrow lesions.

In summary, we present a multi-class diagnosis algorithm to meet the unmet clinical need for early-stage diagnosis and typing of arthritis. Combination of plasma CP, anti-CCP antibody and Hyp gave specific and sensitive detection and discrimination of eOA, eRA, non-RA and good skeletal health, exploiting the discovery of CP in eOA, low levels in healthy people and differential autoimmunity of CP in eOA and eRA.

## Methods

### Patients, healthy subjects and sampling

Patients with longstanding history or established severe, advanced OA (aOA) were recruited at the Department of Rheumatology, Ipswich Hospital NHS Trust, U.K. and Orthopaedic Clinics, University Hospital Coventry & Warwickshire, Coventry, U.K. Patients were undergoing therapeutic knee aspiration and corticosteroid instillation or undergoing total knee replacement due to longstanding symptoms of OA with corresponding radiographic changes. Patients with early-stage OA (eOA) were also recruited at the latter Clinic presenting with new onset knee pain. All patients with eOA had normal radiographs of the symptomatic knee and were undergoing routine exploratory arthroscopy. Macroscopic findings on arthroscopy were classified according to the Outerbridge classification. Patients found to have changes (Outerbridge grade I/II) during routine arthroscopy were recruited for eOA. Patients with advanced rheumatoid arthritis (aRA) were recruited at the Department of Rheumatology, Ipswich Hospital NHS Trust, U.K. and Department of Rheumatology, Royal Devon and Exeter NHS Foundation Trust, Exeter, UK. Patients with early-stage arthritis (within 5 months of the onset of symptoms of inflammatory arthritis) were recruited at the Rapid Access Rheumatology Clinic, City Hospital, Birmingham, U.K. Synovial fluid and peripheral venous blood samples were collected at initial presentation. Diagnostic outcomes were determined at follow-up as non-RA [reactive arthritis (6), pseudogout (1), and unclassified (3)] or early rheumatoid arthritis (eRA). Normal healthy control subjects were recruited according to the following criteria: inclusion criteria - no history of joint symptoms with no arthritic disease or other morbidity; and exclusion criteria - a history of knee injury or knee pain in either knee, taking medication - excepting oral contraceptives and vitamins, and any abnormality at physical examination of the knee. Peripheral venous blood samples from healthy people and subjects with eOA were collected after overnight fasting, as was synovial fluid of eOA study group. Samples were collected in the non-fasted state for eRA, non-RA, aOA and aRA study groups. For analytes studied herein, diurnal variation of serum Hyp had a range of 20%^28^ and plasma CP of healthy subjects was not changed significantly when collected in fasted or non-fasted states (n = 30). Peripheral venous blood samples were collected with EDTA anti-coagulant from patients pre-operatively and synovial fluid obtained intraoperatively from patients, as appropriate. Blood and synovial fluid were centrifuged (2000 g, 10 min) and the resulting plasma and synovial fluid supernatant removed and stored at −80°C until analysis. Synovial fluid was aspirated from the knee joints of patients and stored at −80°C until analysis. Subject characteristics are given in Table 1. Ethical approval for this work was sought and obtained from local ethics committees at Ipswich Hospital NHS Trust, U.K., West Midlands Regional Ethics Committee, U.K., and South West – Exeter Regional Ethics Committee, U.K. The collection of samples from patients and healthy subjects with informed consent and use of them were approved by the local medical ethics committee and were conducted in accordance with the Declaration of Helsinki.

### Analysis of citrullinated protein

The content of citrulline (and arginine) residues in plasma/serum and synovial proteins was quantified in exhaustive enzymatic digests by stable isotopic dilution analysis LC-MS/MS^29^. Plasma or synovial fluid (100 μl) was diluted 5-fold with water and washed by 4 cycles of concentration to 50 μl and dilution to 500 μl with water over a microspin ultrafilter (10 kDa cut-off) at 4°C. The final washed protein (100 μl) was delipidated by extraction 3-times with an equal volume of water-saturated ether. Residual ether was removed in a centrifugal evaporator and protein concentration determined by Bradford method. For enzymatic hydrolysis, an aliquot of protein (100 μg in 20 μl water, free citrulline <62 fmol or <0.002 mmol/mol arg) was mixed with 100 mM HCl (10 μl), pepsin (2 mg/ml in 20 mM HCl; 5 μl) and thymol (2 mg/ml in 20 mM HCl; 5 μl) in HPLC vials with fused glass inserts. The samples were gassed with argon by 3 cycles of placing under vacuum (20 mmHg, 2 min) and re-filling with argon in a centrifugal evaporator. Samples were incubated at 37°C for 24 h. The samples were then neutralized and buffered at pH 7.4 by the addition of firstly 12.5 μl 100 mM potassium phosphate buffer, pH 7.4, and then 5 μl 260 mM KOH. Pronase E (2 mg/ml in 10 mM potassium phosphate buffer, pH 7.4; 5 μl) and penicillin-streptomycin solution (1000 units/ml and 1 mg/ml respectively; 5 μl) was added and the samples were incubated at 37°C for a further 24 h. Finally aminopeptidase (2 mg/ml in 10 mM potassium phosphate buffer, pH 7.4; 5 μl) and prolidase solution (2 mg/ml in 10 mM potassium phosphate buffer, pH 7.4; 5 μl) were added and the samples incubated at 37°C for a further 48 h. All reagents were sterile-filtered, gassed with argon and added automatically by a PAL HTS sample autoprocessor (CTC-PAL Analytics, Zwingen, Switzerland). Protein hydrolysate (25 μl) was spiked with isotopic standards ([^15^N_2_]arg, 5 nmol and [5-^13^C-4,4,5,5-^2^H_4_]citrulline, 25 pmol; 25 μl) and analysed by LC-MS/MS using an Acquity™ UPLC system with a Quattro Premier tandem mass spectrometer (Waters, Manchester, U.K.). Samples are maintained at 4°C in the autosampler during batch analysis. The column was 150 mm × 2.1 mm Hypercarb™ (3 μm particle size; Thermo, Runcorn, U.K.) at 30°C. The mobile phase was 0.1% trifluoroacetic acid (TFA) from 0–5 min and a linear gradient of 0–2.5% acetonitrile from 5–20 min; the flow rate was 0.2 ml/min. Eluate was directed to the mass spectrometer from 4–20 min. Analytes were detected by electrospray positive ionization, multiple reaction monitoring (MRM). The ionization source and desolvation gas temperatures were 120°C and 350°C, respectively. The cone gas and desolvation gas flow rates were 100 and 900 l/h, respectively. The capillary voltage was 3.55 kV. Argon gas (2.7 × 10^−3^ mbar) was in the collision cell. Programmed molecular ion and fragment ion masses optimized to ±0.1 Da and collision energies were and ±1 eV for MRM detection. CP contents of plasma and synovial fluid protein are normalised to arginine content and given as mmol/mol arg.

### Analysis of 4-hydroxyproline

Free Hyp was analysed in plasma and synovial fluid by similar method except 25 μl ultrafiltrate, prepared by 3 kDa cut-off microspin filter with 25 pmol 4,5-[^13^C_2_]Hyp, was analysed. 4,5-[^13^C_2_]Hyp synthesised as described^30^ from [^13^C_2_]glyoxal^31^. Hyp concentrations are given in μM.

### Other assessments

CCP antibody positivity was assessed by automated enzymatic immunoassay (EliA CCP; Phadia, Uppsala, Sweden)^32^.

### Statistical analyses

Data are presented as mean ± SD for parametric distributions and median (lower – upper quartile) for non-parametric distributions. Significance of the difference between means of parametric data was analysed by Student's *t*-test and between medians of non-parametric data were analysed by Mann-Whitney U test for independent samples and Wilcoxon's signed ranks test for paired samples (analytes of plasma and synovial fluid of the same donor). Correlation analysis was performed by the Spearman method. Limit of detection was defined as an analyte amount equivalent to 3 SD of the zero analyte control deduced from response calibration curves. Data were analysed using SPSS, version 16.0. For diagnostic algorithm generation only controls, eOA, eRA and non-RA subject groups were used with data of age, gender, plasma CP, plasma free hydroxyproline, anti-CCP antibodies and RF. For each subject group and method, four “Leave-One-Out” cross-validations were performed and feature selection used to identify which of the features were informative biomarkers. Three different machine learning algorithms were used: Random Forests - a nonlinear, tree-based method^9^, stepwise generalized linear model (GLM) and generalized linear models with elastic net (GLMNET)^10^. The latter uses a sparse form of logistic regression, where it is assumed that parameters for some input variables will be exactly zero (and hence they do not contribute to the analysis). For this we use the R package ‘glmnet'. The 95% CI are determined via bootstrap analysis, using the R package “pROC”^33^. Significance of the 4-class algorithm outcome was assessed by exact binomial test for the null hypothesis that predicted classes are assigned randomly, probability 0.25.

## Author Contributions

U.A., A.A., J.T., N.M., P.J.T. and N.R. acquired the data, U.A., R.S.S., M.L.C., A.F., K.R., R.A.W., P.G.W., R.C.H., P.J.T. and N.R. analysed and interpreted the data, U.A., R.S.S., P.J.T. and N.R. drafted the report and all authors reviewed and revised it critically and approved the final version to be published.

## Supplementary Material

Supplementary Information Supporting information

## Figures and Tables

**Table 1:** Clinical characteristics of healthy people and patients with arthritis

Analysis group	Subject group	N	Age (yr)	Gender (M/F)	Anti-CCP antibodies (+/−)	ESR (mm/h)	CRP (mg/L)	Smoking (+/−)	Duration of disease (yr)
Training set	Control	16	50 ± 8	9/7	ND	ND	ND	1/14	-----------
	non-RA	10	37 ± 12**	8/2	0/10	25 (16–24)	38 (7–85)	3/7	0.1 (0.1–0.2)
	eRA	10	65 ± 11***,OOO	4/6	7/3	68 (27–21)^O^	65 (37–12)	8/2	0.2 (0.1–0.3)
	aRA	22	60 ± 15 *,OOO	8/14	22/0	32 (17–15)	24 (8–36)	ND	7 (3–20) OOO, †††
	eOA	16	48 ± 8 OO	9/7	0/16	ND	2.1 (1.9–1.6)	1/12	0.3 (0.1–1.5)
	aOA	17	69 ± 9***,OOO,†††	7/11	ND	ND	4.3 (2.6–3.6)	1/13	2 (2–7) OOO, †††
Test set	Control	37	34 ± 9	17/20	0/37	ND	0.21 (0.15–0.29)	0/37	-----------
	non-RA	32	52 ± 18***	14/16	0/32	17 (10–34)	14 (6–27) **	9/23	0.1 (0.04–0.23)
	eRA	35	60 ± 16***	13/22	18/17	24 (6–23)^O^	19 (11–41)***	6/21	0.2 (0.04–0.24)
	eOA	30	46 ± 14**OOO	13/17	0/30	ND	0.62 (0.55–1.46)	3/24	0.3 (0.1–1.5)

Significance: *, ** and ***, *P* > 0.05, *P* < 0.01 and *P* < 0.001 with respect to control (healthy people); ^OO^ and ^OOO^, *P* < 0.01 and *P* < 0.001 with respect to non-RA; and †††, P < 0.001 for aRA with respect to eRA and for aOA with respect to eOA. Data are mean ± SD (parametric) and median [lower – upper quartile] (non-parametric). ND, not determined.

**Table 2:** Multiclass algorithm outcome with test set cohort using the GLMNET algorithm

	Control	eOA	eRA	non-RA
nCorrect	15/37	22/30	20/35	8/32
Sensitivity	0.41 (0.25–0.58)	0.73 (0.54–0.88)	0.57 (0.39–0.74)	0.25 (0.11–0.43)
Specificity	0.75 (0.65–0.83)	0.87 (0.78–0.92)	0.91 (0.83–0.96)	0.76 (0.68–0.85)
F-Measure	0.54	0.78	0.68	0.36

Data are 95% CI for sensitivity and specificity given in brackets. Predictive performance of the algorithm trained using the training data set and predicting for the test data set. We emphasise ambitious nature of a multiclass analysis of this type. In order to correctly identify a given case, we must discriminate between three additional, potentially very different conditions, as opposed to just one (in the standard classification case). For example, if one were to assign cases at random to disease groups, one would only achieve a 25% success rate. The above predictive performance should be considered with this in mind.

**Table 3:** Confusion matrix for GLMNET multiclass algorithm

		Predicted class			
		Control	Early OA	Early RA	non-RA
Clinical class	Control	15	3	9	12
	eOA	3	22	4	5
	eRA	1	1	20	7
	non-RA	18	4	2	8
