# Supplementary material for: Biomarkers of early stage osteoarthritis, rheumatoid arthritis and musculoskeletal health
Source: Sci Rep. 2015 Mar 19;5:9259. doi: 10.1038/srep09259 (PMC4365413; doi:10.1038/srep09259)
Supplement: Supplementary Information — Supporting information [file srep09259-s1.doc]

**Supporting Information**

**Biomarkers of early stage osteoarthritis,** **rheumatoid arthritis and musculoskeletal health**

Running title: Citrullination and differential autoimmunity in early arthritis

Usman Ahmed,^1^ Attia Anwar,^1^ Richard S Savage,^1,2^ Matthew L. Costa**,**^1,9^ Nicola Mackay,^1^ Andrew Filer,^3,4^ Karim Raza,^3,4^ Richard A. Watts,^5,6^ Paul G. Winyard,^7^ Joanna Tarr, ^7^ Richard C. Haigh,^7,8^ Paul J. Thornalley,^1,2^ and Naila Rabbani^1,2^

^1^Warwick Medical School, Clinical Sciences Research Laboratories, University of Warwick, University Hospital, Coventry CV2 2DX, U.K.; ^2^Warwick Systems Biology Centre, Coventry House, University of Warwick, Coventry CV4 7AL, U.K.; ^3^Sandwell and West Birmingham Hospital NHS Trust, Dudley Road, Birmingham B18 7QH, West Midlands, U.K.; ^4^Centre for Translational Inflammation Research, University of Birmingham, Birmingham B15 2TT, U.K., ^5^Ipswich Hospital NHS Trust, Ipswich IP4 5PD, Suffolk, U.K., ^6^Medical School, University of East Anglia, Norwich, NR4 7TJ UK, ^7^University of Exeter Medical School, St Luke’s Campus, Exeter EX1 2LU, U.K., and ^8^Department of Rheumatology, Royal Devon and Exeter NHS Foundation Trust, Exeter, UK. ^9^Warwick Clinical Trials Unit, University of Warwick, Coventry CV4 7AL, U.K.

Corresponding author: Naila Rabbani [n.rabbani@warwick.ac.uk](mailto:n.rabbani@warwick.ac.uk)

**Supporting text S1 | Effect of drug therapy on CP and free hydroxyproline.** The effect of drug therapy on CP and Hyp in patients with aRA was evaluated. Patients receiving anti-TNFα therapy had lower plasma Hyp with respect to those not receiving anti-TNFα therapy (0.96 µM versus 3.37 µM, *P*<0.01). Patients receiving treatment with non-steroidal anti-inflammatory drugs (NSAIDs) had lower synovial fluid CP (0.15 versus 1.02 mmol/mol arg, *P*<0.05) and plasma Hyp (0.99 µM versus 3.16 µM, *P*<0.01) with respect to those not receiving NSAIDs. However, patients receiving treatment with prednisolone had higher synovial fluid CP (1.72 versus 0.24 mmol/mol arg, *P*<0.01) and plasma Hyp (2.87 versus 0.94 µM, *P*<0.05) with respect to those not receiving prednisolone. Treatment with or without methotrexate and opiate analgesics was not associated with differences in these variables.

**Table S1 | Analytical variables and assay characteristics for quantitation of citrulline, arginine and 4-hydroxyproline by stable isotopic dilution analysis tandem mass spectrometry.**

| Analyte | Citrulline | Arginine | 4-Hydroxyproline |
| --- | --- | --- | --- |
| Retention time R_t_ (min) | 13.5 | 16.5 | 5.6 |
| Molecular ion M+1 (Da) | 176.1 | 175.1 | 132.0 |
| Fragment ion (Da) | 70.1 | 70.1 | 86.1 |
| Cone voltage (V) | 20 | 30 | 26 |
| Collision energy (eV) | 21.0 | 24.0 | 12.0 |
| Neutral fragment losses | H_2_CO_2_, NH_2_C(=O)NH_2_ | H_2_CO_2_, NH_2_C(=NH)NH_2_ | H_2_CO_2_ |
| Internal standard | [5-^13^C,4,4,5,5-^2^H_4_]citrulline | [guanidino-^15^N_2_]arg | 4,5-[^13^C_2_]Hydroxyproline |
| Limit of detection (fmol) | 62 | 520 | 102 |
| Intra- and interbatch CV (%; n = 6) | 1.3 and 6.0 | 1.0 and 1.8 | 1.0 and 1.5 |
| Recovery (%) | 88 (protein digest) | 94 (protein digest) | 100 (ultrafiltrate) |

**Table S2 |** Cross validation of multiclass algorithm. Improved outcome with training set and test set cohorts combined using GLMNET algorithm and randomly assigning 67% samples to training set and 33% to the test set.

**A. Multi-class analysis for GLMNET algorithm**

|  | Control | eOA | eRA | non-RA |
| --- | --- | --- | --- | --- |
| nCorrect | 13/17 | 10/14 | 10/15 | 7/15 |
| Sensitivity | 0.76 (0.50 – 0.93) | 0.71 (0.42 – 0.92) | 0.67 (0.38 – 0.88) | 0.47 (0.21 – 0.72) |
| Specificity | 0.77 (0.62 – 0.89) | 0.96 (0.85 – 0.99) | 1.00 (0.92 – 1.00) | 0.80 (0.66 – 0.91) |
| F-Measure | 0.65 | 0.77 | 0.80 | 0.45 |

**B. Confusion matrix for GLMNET algorithm**

|  | | Predicted class | | | |
| --- | --- | --- | --- | --- | --- |
|  |  | Control | Early OA | Early RA | non-RA |
| Clinical  class | Control | 13 | 2 | 0 | 8 |
|  | eOA | 2 | 10 | 0 | 0 |
|  | eRA | 0 | 0 | 10 | 0 |
|  | non-RA | 2 | 2 | 5 | 7 |


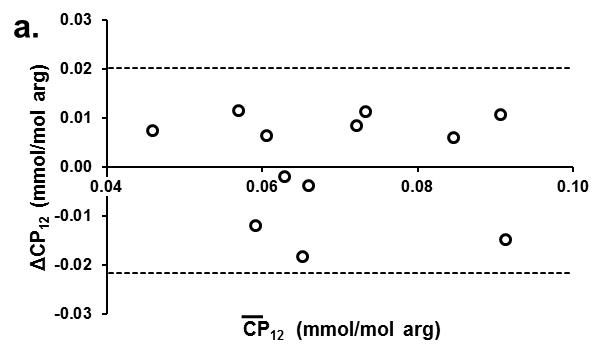


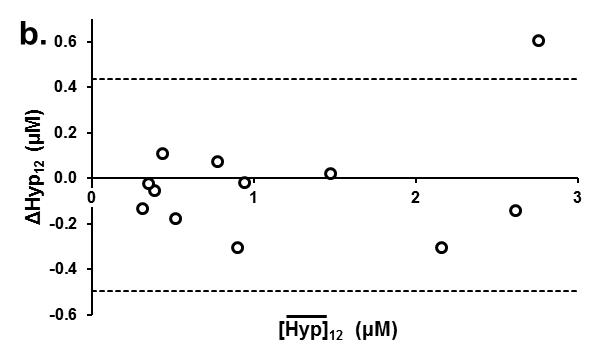


**Figure S1 |** Bland-Altman plots for (a) CP and (b) Hyp measurements. Data from samples analysis of 12 subjects (7 healthy controls and 5 eOA) was evaluated. There was no proportional bias but one outlier was identified in the Hyp measurements.
